# 5-HT1A/1B Receptors as Targets for Optimizing Pigmentary Responses in C57BL/6 Mouse Skin to Stress

**DOI:** 10.1371/journal.pone.0089663

**Published:** 2014-02-21

**Authors:** Hua-Li Wu, Si-Lin Pang, Qiong-Zhen Liu, Qian Wang, Min-Xuan Cai, Jing Shang

**Affiliations:** New Drug Screening Center, China Pharmaceutical University, Nanjing, China; University of Tennessee, United States of America

## Abstract

Stress has been reported to induce alterations of skin pigmentary response. Acute stress is associated with increased turnover of serotonin (5-hydroxytryptamine; 5-HT) whereas chronic stress causes a decrease. 5-HT receptors have been detected in pigment cells, indicating their role in skin pigmentation. To ascertain the precise role of 5-HT in stress-induced pigmentary responses, C57BL/6 mice were subjected to chronic restraint stress and chronic unpredictable mild stress (CRS and CUMS, two models of chronic stress) for 21 days, finally resulting in abnormal pigmentary responses. Subsequently, stressed mice were characterized by the absence of a black pigment in dorsal coat. The down-regulation of tyrosinase (TYR) and tyrosinase-related proteins (TRP1 and TRP2) expression in stressed skin was accompanied by reduced levels of 5-HT and decreased expression of 5-HT receptor (5-HTR) system. In both murine B16F10 melanoma cells and normal human melanocytes (NHMCs), 5-HT had a stimulatory effect on melanin production, dendricity and migration. When treated with 5-HT in cultured hair follicles (HFs), the increased expression of melanogenesis-related genes and the activation of 5-HT1A, 1B and 7 receptors also occurred. The serum obtained from stressed mice showed significantly decreased tyrosinase activity in NHMCs compared to that from nonstressed mice. The decrease in tyrosinase activity was further augmented in the presence of 5-HTR1A, 1B and 7 antagonists, WAY100635, SB216641 and SB269970. *In vivo*, stressed mice received 5-HT precursor 5-hydroxy-l-tryptophan (5-HTP), a member of the class of selective serotonin reuptake inhibitors (fluoxetine; FX) and 5-HTR1A/1B agonists (8-OH-DPAT/CP94253), finally contributing to the normalization of pigmentary responses. Taken together, these data strongly suggest that the serotoninergic system plays an important role in the regulation of stress-induced depigmentation, which can be mediated by 5-HT1A/1B receptors. 5-HT and 5-HTR1A/1B may constitute novel targets for therapy of skin hypopigmentation disorders, especially those worsened with stress.

## Introduction

Stress is a complex state of threatened homeostasis, which mobilizes a composite spectrum of adaptive physiological and behavioral responses to restore and maintain challenged homeostasis [1]. It is well-known that the skin is the largest body organ acting as a biological barrier separating the internal milieu from noxious external environmental factors to maintain local and systemic homeostasis [2], [3]. Its functions are integrated into the skin immune, pigmentary, epidermal and adnexal systems, and are in continuous communication with the systemic immune, neural and endocrine systems [2]–[7]. The skin-derived systems can activate cutaneous nerve endings to alert the brain on changes in the epidermal or dermal environments, or to alternatively activate other coordinating centers by direct (spinal cord) neurotransmission without brain involvement [8]. Skin melanocytes can also secrete classic stress neurotransmitters, neuropeptides and hormones, and express corresponding receptors to organize a regulatory network for the maintenance of cutaneous homeostasis [7]. Melanocytes are located in three separate compartments in the skin: the epidermis, the dermis and the hair follicles. Some pathways regulating melanocytes are specific to hair follicle (HF) or skin [1]. Hair follicle has its own specific cycle which is defined as rapid growth (anagen), regression (catagen) and resting periods (telogen) according to histological morphology [9]–[11]. Up to date, numerous clues have revealed that stress plays an important role in abnormal hair cycling and pigmentation [12]–[14].

Stress response includes activation of the HPA axis and the autonomic nervous system, both of which interact with the immune system, activating skin mast cells, macrophages, keratinocytes and T lymphocytes [1]. Experimental and clinical researches have showed that stress also initiates a cascade of changes with impact on the 5-HT system. Acute stress is associated with an increase in the turnover of 5-HT, whereas chronic stress with a sustained increase in plasma cortisol, causes a reduction in serotonin turnover and release [15]. Conversely, under stressful conditions, brain 5-HT is shown to regulate synthesis of adrenocorticotropic hormone (ACTH) and cortisol [16], [17]. Serotonin (5-hydroxytryptamine; 5-HT) has many physiological effects on a variety of organs, such as blood pressure regulation, stress response, appetite and memory [18]. It acts via seven different classes of serotonergic receptors (5-HTR1-7) with at least 21 subtypes [19]. Of these receptors 5-HT1A shows a decreased responsiveness during chronic mild stress [20], [21], while the 5-HT2AR affinity for 5-HT is increased by chronic stress [22].

The mammalian skin cells have the capability to produce and metabolize serotonin. The cutaneous phenotypic effects are mediated by its interactions with 5-HT receptors [23]–[26]. 5-HT receptors are widely detected on mammalian melanocytes and dermal fibroblasts [27]. Accordingly, there has been a complex relationship between skin pigmentary function and 5-HT. For example, 5-HT dose-dependently inhibits melanin production and tyrosinase activity in human SK-MEL-188 melanoma cells [28]. 5-HT stimulates proliferation of melanocytes in a medium deprived of growth factors, whereas it inhibits cell growth in the presence of growth factors [29]. In other species including lizards, 5-HT has positive effects on melanogenesis [30]. Moreover, 5-HT induces melanogenesis via stimulation of 5-HT2R in SK-MEL-2 melanoma cells [31]. Fluoxetine (FX), a member of the class of selective serotonin reuptake inhibitors, can also lead to a dramatic increase in melanin production in both murine B16F10 cells and NHMCs [31], [32]. *In vivo,* 5-HT is also best known to have various roles in skin, e.g. pro-edema, vasodilatory, pro-inflammatory and pruritogenic [23]. Earlier, treatment with 5-HT2AR antagonists reduced the severity of contact allergic reactions in mice [33]. Tandospirone, an agonist of 5-HT1AR, reduces the stress level and attenuates itching in patients with atopic dermatitis [34]. The content of 5-HT in blood is decreased in patients with vitiligo as compared with healthy persons [35]. Emerging evidence suggests a role for 5-HT signaling in controlling the development of a number of skin diseases, including hypopigmentation. However, molecular mechanisms of 5-HT-led cutaneous pigmentary disorders in stress remain poorly understood.

Thus, this current study aims to explore the possible role of 5-HT system in the pigmentation function in response to stress. We used two types of stressed-mice, namely chronic restrain stress (CRS) and chronic unpredictable mild stress (CUMS). The skin truncal melanocytes in mice are confined to the hair follicle and the intrafollicular melanogenesis exclusively reflects the skin color [36], [37]. According to the strict coupling of follicular melanogenesis and HF cycling, anagen development is associated with special changes in skin pigmentation. In catagen, melanin formation is switched off and is absent during telogen. Therefore, we mainly tested the melanin synthesis of hair follicles during the development of depilation-induced anagen (days 0 = telogen, and days 1–12, after anagen induction).

## Materials and Methods

### Animals

All experiments were approved according to the Animal Experimentation Ethics Committee of the Chinese Pharmaceutical University (Approval ID: SCXK- (Jun) 2004-004) and performed in strict accordance with the guidelines of the “Principles of Laboratory Animal Care” (NIH Publication No.80-23, revised in 1996). Adult male C57BL/6 mice (8∼10 weeks old, weighing 25–30 g) were obtained from the Laboratory Animal Service Center of Yangzhou University. All animals were acclimated for one week under the following conditions: the room temperature was 23±1°C; humidity was 50±5% with a 12-hour light/dark cycle (lights on at 6∶00 a.m. and off at 6∶00 p.m.). During this period, food and water were provided *ad libitum*.

### Animal Experimental Design and Anagen Induction

Two types of stress, namely chronic restrain stress (CRS) and chronic unpredictable mild stress (CUMS), were imposed on mice. Five mice (Control group) were housed per cage for 21 days. There were 15 mice in every group. According to the reported method, mice (CRS group) were restrained daily for 6 h (10∶00 a.m.–16∶00 p.m.) before blood and skin samples were collected on day 21 [38]. Chronic unpredictable mild stress protocol was adapted from Gamaro et al. [39]. One of the following stressors was carried out each day (Table 1): (1) 24 h of damp bedding; (2) light during the night; (3) 1–3 h of restraint; (4) 24 h of home cage inclination at an angle of 45°; (5) 5 min of ice swimming; (6) 24 h of food deprivation; (7) 24 h of water deprivation; (8) 1 h of foot shock (the current 0.6 mA, two times a minute). Stress was applied at different schedules every day, in order to increase unpredictability.

**Table 1:** Schedule of stressor agents used during chronic unpredictable mild stress.

Days	Stressors	Days	Stressors
1	3 h of restraint	12	24 h of water deprivation
2	Light during the night	13	5 min of ice swimming
3	24 h of food deprivation	14	3 h of restraint
4	24 h of damp bedding	15	Light during the night
5	1 h of foot shock	16	24 h of home cage inclination
6	24 h of water deprivation	17	24 h of food deprivation
7	24 h of home cage inclination	18	1 h of foot shock
8	5 min of ice swimming	19	5 min of ice swimming
9	depilation	20	24 h of damp bedding
10	1 h of foot shock	21	3 h of restraint
11	Light during the night		

On the 9th day of two types of stress, we performed procedures of depilation to induce anagen of hair cycle as described previously [12]. In brief, wax/rosin mixture (1∶1 on weight) was applied to the dorsal skin (from neck to tail) of C57BL/6 mice with all HFs in telogen. Peeling-off the wax/rosin mixture removed all hair shafts and immediately caused homogeneous anagen development over the entire depilated back area, thus inducing a highly synchronized anagen development.

### Drugs

5-Hydroxytryptophan (5-HTP), CP 94253 (a selective serotonin 5-HT1B receptor agonist) and 8-OH-DPAT (a selective serotonin 5-HT1A receptor agonist) were purchased from Sigma-Aldrich (St Louis, MO, USA). Drugs dissolved in saline were injected intraperitoneally except for fluoxetine (FX). FX (Academy of Food and Drug Identification, China) was transdermally delivered using pluronic lecithin organogels [40] and microemulsions [41], [42] as have been reported. The concentration of FX was 20 mg/mL. Transdermal delivery of FX can be an effective strategy for avoiding gastrointestinal irritation and reducing side-effects from fluctuations in plasma concentration [41]. Mice were randomly divided into the following eleven groups: (1) Control group; (2) CRS group: no drug injection and application of CRS; (3) CUMS group: no drug injection and application of CUMS; (4) R5-HTP group: application of CRS concomitant with 100 mg/kg/day of 5-HTP injection intraperitoneally for 12 days [43]; (5) M5-HTP group: application of CUMS concomitant with 100 mg/kg/day of 5-HTP injection intraperitoneally for 12 days; (6) RFX group: application of CRS concomitant with fluoxetine delivery transdermally for 12 days [41]; (7) MFX group: application of CUMS concomitant with fluoxetine delivery transdermally for 12 days; (8) R5-HT1A group: application of CRS concomitant with 0.5 mg/kg/day of 8-OH-DPAT injection intraperitoneally for 12 days [44]; (9) M5-HT1A group: application of CUMS concomitant with 0.5 mg/kg/day of 8-OH-DPAT injection intraperitoneally for 12 days; (10) R5-HT1B group: application of CRS concomitant with 5 mg/kg/day of CP 94253 injection intraperitoneally for 12 days [45]; (11) M5-HT1B group: application of CUMS concomitant with 5 mg/kg/day of CP 94253 injection intraperitoneally for 12 days.

### Other Reagents

5-HT hydrochloride (≥99% HPLC solid) was purchased from Sigma Aldrich (St Louis, MO, USA). Dimethylsulfoxide (DMSO), L-3, 4-dihydroxyphenylalanine (L-DOPA), 3-isobutyl-1-methylxanthine (IBMX), phorbol esters (TPA), cholera toxin (CT), and horseradish peroxidase-conjugated secondary antibodies were purchased from Sigma-Aldrich (MO, USA). Enhanced BCA protein assay kit, phenylmethylsulfonyl fluoride (PMSF) and cell lysis buffer for Western Blot were from Beyotime Institute of Biotechnology (China). Total protein extraction kit was from Applygen Technologies Inc. (China). Other reagents were of the highest quality available.

### Tissue and Serum Preparation

On day 12 after depilation when the hair cycle of control mice was in the late anagen VI [10], [11], blood samples (500 µL) were collected from enucleated eyeball of mice under intraperitoneal anesthesia with chloral hydrate (300 mg/kg), and centrifuged at 1500 g for 10 min at 4°C. Serum samples from the centrifuging process were transferred to Eppendorf tubes and stored at −80°C until analyzed [46]. Skin specimens from depilated back were harvested about 2×5 cm to obtain longitudinal sections through the hair units, which was an essential requirement for the histomorphology of hair pigmentation [12]. They were partially fixed in 4% paraformaldehyde then embedded in paraffin-wax. The remaining skin specimens were wrapped in aluminium foil, deep frozen in liquid nitrogen.

### Measurement of 5-HT Levels in Serum and Skin

Firstly, sample preparation procedure was carried out. 4.0 mL water (containing 0.1% formic acid methanol) was added to 1.0 g of mice skin to prepare tissue homogenate. The mixture was vortexed and centrifuged at 16000 rpm for 5 min. The supernatant (580 µL) was spiked with 10.0 µL of 3,4-dihydroxy benzyl amine (10 µg/ml) and vortexed for 3 min. The supernatant (420 µL) was evaporated to dryness under 40°C water bath for a stream of nitrogen after centrifugation at 16000 rpm for 5 min. 100 µL water (containing 0.2% formic acid and 0.1% ammonium acetate) was added to the residue and vortexed under room temperature for 3 min, then 200 µL chloroform-isopropyl alcohol (10∶3 v/v) was added to the mixture and vortexed for 3 min. After centrifugation at 16000 rpm for 5 min, 10 µL of supernatant was injected into an liquid chromatography-tandem mass spectrometry (LC-MS/MS) system for analysis.

Serum samples (100 µL) were spiked with 10 µL of 3,4-dihydroxy benzyl amine (10 µg/ml) and the mixtures were vortexed for 1 min before precipitation with 200 µL 0.1% formic acid acetonitrile. The mixture was vortexed for 3 min followed by centrifugation at 16000 rpm for 5 min. The supernatant (200 µL) was then evaporated to dryness under 40°C water bath for nitrogen. 50 µL water (containing 0.2% formic acid and 0.1% ammonium acetate) was added to the residue and vortexed under room temperature for 3 min. After centrifugation at 16000 rpm for 5 min, 10 µL of supernatant was injected for analysis.

The LC-MS/MS system was composed of an Agilent 1100 HPLC system (Agilent Technologies, Inc., USA) and a Finnigan Surveyor LC-TSQ Quantum Ultra AM mass spectrometer, Xcalibur 1.2 software for data acquisition and analysis (Thermo Finnigan, San Jose, CA, USA). The analytical column acquired from Hanbon Sci. & Tech. (Jiangsu, China) was an Hanbon Lichrospher C18 (4.6 mm×25 cm, 5 µm) and the temperature was maintained at 30°C. The following gradient elution with acetonitrile as “A” and 0.1% aqueous ammonium acetate, 0.2% formic acid as “B” was used at a flow rate of 1000 µL/min: 0–6 min: 2% A→8% A; 6–8 min: 8% A → 70% A; 8–8.01 min: 70% A →2% A and 8.01–12 min: 2% A. The sample injection volume was 10 µL for all analyses.

The mass spectrometer was operated in the positive electrospray ionization mode with the spray voltage set at 5.0 kV. Nitrogen sheath gas pressure was set at 40 Arb, auxiliary gas at 10 Arb. The vaporizer temperature was set at 350°C. The collision energy of 8eV was used with argon at a pressure of 1.5 m Torr for collision-induced dissociation (CID). Quantification was performed with selected reaction monitoring (SRM) of the transitions of m/z 177.1→160 for 5-HT and 140.0→123.0 for 3, 4-dihydroxy benzyl amine (internal standard) with a scan time of 0.4 s per transition.

### Employment of 5-HT Receptor Antagonists

To further characterize the direct effect of 5-HT receptor on murine B16F10 melanoma cells, various antagonists were tested for their ability to inhibit serotonin-induced melanogenesis (Table 2). The antagonist was applied to the incubating media before the addition of 100 µM serotonin. The final concentration of antagonists was selected for 10 µM, except for 5-HT1B and 5-HT5A antagonists at 1 µM [31], [47].

**Table 2:** Summary of the effects of drugs used in this study on melanin synthesis within B16F10 cells.

Drug	Pharmacology	Receptor efficacy	Effect on Melanogenesis
5-HT hydrochloride	Stimulation of 5-HT receptor	Non-selective endogenous agonist	+
WAY100635	Blockage of 5-HT_1A_ receptor	Highly selective 5-HT_1A_ antagonist	−
SB216641	Blockage of 5-HT_1B_ receptor	Selective 5-HT_1B_ antagonist	−
BRL15572	Blockage of 5-HT_1D_ receptor	Selective 5-HT_1D_ antagonist	None
Ketanserin	Blockage of 5-HT_2A_ receptor	Selective 5-HT_2A_ antagonist	−
SB699551	Blockage of 5-HT_5A_ receptor	Selective 5-HT_5A_ antagonist	−
SB269970	Blockage of 5-HT_7_ receptor	Selective 5-HT_7_ antagonist	−

### Assessment of Hair Cycle and Hair Pigmentation

All mice were photographed with a digital camera (Canon, Japan) once every day after depilation. The grayscale (0–255) of specific area in the photographs (the region from neck to tail) were analyzed by Image J software and presented as ratios (grayscale/255). The HE stain was used to quantify the stage of the hair follicles using a published classification technique based on the morphology of the dermal papilla and sebaceous glands [10]. Then, hair cycle score was assessed as described previously [11], [13]. In addition, the melanin granule in HFs was visualized histochemically.

### Effect of 5-HT on 5-HTRs Activation in HFs

Microdissected anagen VI HFs were obtained from C57BL/6 mice vibrissaes. Mice vibrissae HFs were cultured in a supplemented, serum-free culture medium for 7 days [11], [48], with the addition of serotonin 10–1000 µM [31] or culture medium only. After 5-HT-treatment for 7 days, mice vibrissae HFs were harvested for Q-PCR assay.

### RT-PCR

RNA was extracted using TRIzol (Invitrogen) and first-strand cDNA synthesis was performed using Advantage RT-for-PCR (TaKaRa, China). For conventional reverse transcription-polymerase chain reaction (RT-PCR) cDNA was amplified using Taq DNA polymerase (TaKaRa, China). Comparable quantities of cDNA were ensured by amplification of GAPDH. Primers and cycling conditions for GAPDH, 5-HT1A, 5-HT1B, 5-HT1D, 5-HT1F, 5-HT2A, 5-HT2B, 5-HT2C, 5-HT3, 5-HT4, 5-HT5A, 5-HT5B, 5-HT6, and 5-HT7 were applied as reported previously [49]. PCR products were resolved as single bands by agarose gel electrophoresis and visualized with nucleic acid dye (GoldView). The expression levels were assessed by an image analysis system. For quantitative PCR, cDNA was amplified using iQ SYBR Green Supermix (TaKaRa, China) in an MJ Research Chromo4 System (Bio-Rad Laboratories). All reactions were performed in triplicate, with cycling conditions as for conventional RT-PCR.

Expression of MITF, TYR, TRP1 and TRP2 was assessed by using quantitative PCR. Primers for these four genes were as follows: MITF (forward, 5–TGC TCG CCT GAT CTG GTG AAT-3, and reverse, 5-GTG CCG AGG TTG TTG GTA AAG G-3), TYR (forward, 5–GAT GGA ACA CCT GAG GGA CCA CTA T-3, and reverse, 5-GCT GAA ATT GGC AGT TCT ATC CAT T-3), TRP1, (forward, 5–CGCACCTATTGGACATAACAGGC-3, and reverse, 5-ACA ACG CAG CCA CTA CAG CAA T-3), TRP2, (forward, 5–CAG AAA TAA TGA GAA ACT GCC AAC C-3, and reverse, 5-AGT CCA GTG TTC CGT CTG CTT TAT C-3). Transcripts were all amplified by 40 cycles of the following: 95°C for 30 s (denaturation), 60°C for 30 s (annealing) and 72°C for 30 s (extension).

### Western Blot

The dorsal skin was quickly dissected out and then lysed in 400 µL RIPA buffer (50 mM Tris-HCl (pH 7.4), 150 mM NaCl, 1 mM PMSF, 1 mM EDTA, 1% Triton X-100, 0.5% sodium deoxycholate, and 0.1% SDS). After centrifugation at 12.000 rpm/min for 20 min at 4°C, 20 µg of total protein of each sample was loaded into a 12% SDS-PAGE gel and then transferred to PVDF membranes (Millipore). The membrane was blocked with 5% non-fat dry milk in TBS containing 0.05% Tween-20 (TBS-T) for 1 h and incubated with goat polyclonal antibodies against TYR (Product number SC7833), TRP1 (Product number SC10443), rabbit polyclonal antibodies against TRP2 (Product number AB74073, 1∶1000, Abcam, Cambridge, UK), mouse polyclonal antibodies against β-actin (Product number CST3700, 1∶1000, Cell Signaling Technology Inc., MA, USA). After reaction with the second antibody, proteins were visualized by an enhanced chemiluminescence detection system. Densitometric analysis was again carried out by using the Quantity One (Bio-Rad) to scan the signals. Western blot assay results were representative of at least 3 independent experiments.

### Cell Culture

The murine melanoma cell line B16F10 was purchased from the Cell Bank of the Chinese Academy of Sciences, Shanghai, China and maintained as a monolayer culture in Dulbecco’s Modified Eagle’s Medium (DMEM; Gibco/Invitrogen, Carlsbad, CA) supplemented with 10% (v/v) heat-inactivated fetal bovine serum (FBS; Gibco/Invitrogen), 100 U/ml penicillin, 100 µg/ml streptomycin (Gibco/Invitrogen), at 37°C in a humidified incubator with 5% CO_2_.

The studies on human material were approved by Nanjing Drum Tower Hospital, Medical Ethics Committee. All participants provided their written informed consent, which was approved by the Nanjing Drum Tower Hospital, Medical Ethics Committee. Normal human foreskin-derived epidermal melanocytes (NHMCs) were from young male adult foreskins (ethnic Han/aged 18–22 years) obtained at circumcision following standard protocols [50]. Briefly, foreskins were cut into strips and digested with 0.25% trypsin at 4°C for about 20 h. After digestion, epidermis was separated from dermis. The NHMC suspension was filtered and cells were washed twice at 1500 rpm for 6.5 min prior to resuspension in MCDB153 medium (Sigma), supplemented with IBMX, TPA, CT and 100 U/ml penicillin and 100 µg/ml streptomycin (GIBCO, USA). NHMCs were cultured in a humidified atmosphere with 5% CO_2_ at 37°C.

### Assay of Melanin Content in B16F10 Cells and NHMCs

Melanin content was measured as described previously with minor modification [51]. Cells were treated with 5-HT for 48 h and then harvested by centrifugation. Total melanin in the cell pellet was dissolved in 100 µL of 1 N NaOH/10% DMSO for 1 h at 80°C and solubilized melanin was measured at 405 nm using the µQuant microplate reader.

### Transwell Migration Assay

The bottom chambers of Transwell were filled with MCDB153 (0.5% FBS) or DMEM (2.5% FBS) supplemented with different concentrations of 5-HT, and the top chambers were seeded inactivated 5×10^4^ cells/well NHMC in 200 µL MCDB153 (0.5% FBS) or 10×10^4^ cells/well B16F10 in 200 µL DMEM (2.5%). After 24 h of migration, the cells on the top surface of the membrane (nonmigrated cells) were scraped and the cells spreading on the bottom sides of the membrane (migrated cells) were fixed with 4% paraformaldehyde for 30 min. Thereafter, those migrated cells were stained with 0.1% hexamethylpararosaniline. Images were taken by Olympus inverted microscope and migrated cells were quantified by manual counting.

### Assessment of Morphological Change

The cells were seeded in a six-well plate in DMEM or MCDB153 supplemented with 2.5% fetal bovine serum (FBS). After 72 h of incubation, the morphological changes were observed by optical microscopy.

### Tyrosinase Activity

Tyrosinase activity was determined using L-DOPA after treatment with 5-HTR antagonists or 10% of the serum obtained from stressed mice as described by Tomita et al [52]. Normal human melanocytes were plated at a density of 10000 per well in 100 µL of medium in 96-well plates and cultured for 3 d. The cells were washed with phosphate-buffered saline and lysated with 45 µL of 1% Triton X-100, and then incubated with 1 mM L-DOPA for 1 h at 37°C. The absorbance was measured at 475 nm with a spectrophotometer.

### Statistical Analysis

All data were represented as mean ± SD. Statistical analysis of results was performed using one-way ANOVA with Tukey’s correction for multiple comparisons. All data were analysed using GraphPad Prism software (UK). *P*<0.05 was considered significant.

## Results

### *In vivo* Effect of Stress on Pigmentary Responses in C57BL/6 mice

**Figure 1:**
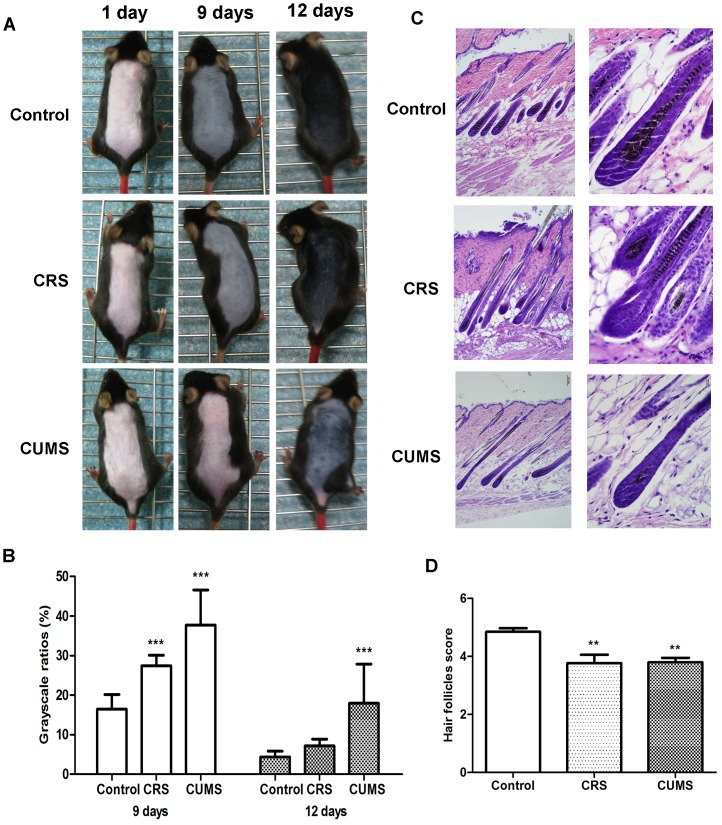
Macroscopic observations of the pigmentary response and the hair cycle stage after stress. A: The significant area of color in the dorsal skin was from neck to tail. B: The corresponding skin color gray-scale ratio on day 9 was shown on the left and day 12 on the right. C: A representative area of each group on day 12 after depilation with the majority of hair follicles. Original magnification was ×100 on the left and ×400 on the right. D: The results of hair follicles score for day 9 were shown on the left and the data for day 12 on the right. For each mouse a minimum of 6 individual visual fields were assigned to define hair cycle stages. Data are presented as mean ± SD, n = 15 in each group, *P<0.05, **P<0.01 and ***P<0.001 vs control group.

To ascertain whether stress influences hair pigmentation, CRS or CUMS were imposed on mice as described above. On days 9 and 12 after depilation, stressed mice showed obvious whitening of the dorsal skin (Figure 1A). In contrast to CUMS mice, CRS mice showed progressive darkening of the dorsal coat (Figure 1A). Also, black pigment was seen in nonstressed mice (Figure 1A). Meanwhile, the corresponding skin grayscale ratio in control mice was significantly lower than that in both CRS mice and CUMS mice (Figure 1B). On day 12, most of hair follicles in control mice entered catagen or anagen-catagen transition and the majority of hair follicles in stressed mice were still in anagen IV-VI (Figure 1D). In addition, on days 9 and 12, morphological observations revealed a decreased amount of histochemically detectable melanin granules in HFs of stressed mice compared with nonstressed mice (Figure 1C). These results suggest that two types of stress exert inhibitory effects on hair pigmentation.

### Effect of Stress on Transcription and Expression of Tyrosinase and Tyrosinase-related Proteins in the Dorsal Skin

**Figure 2:**
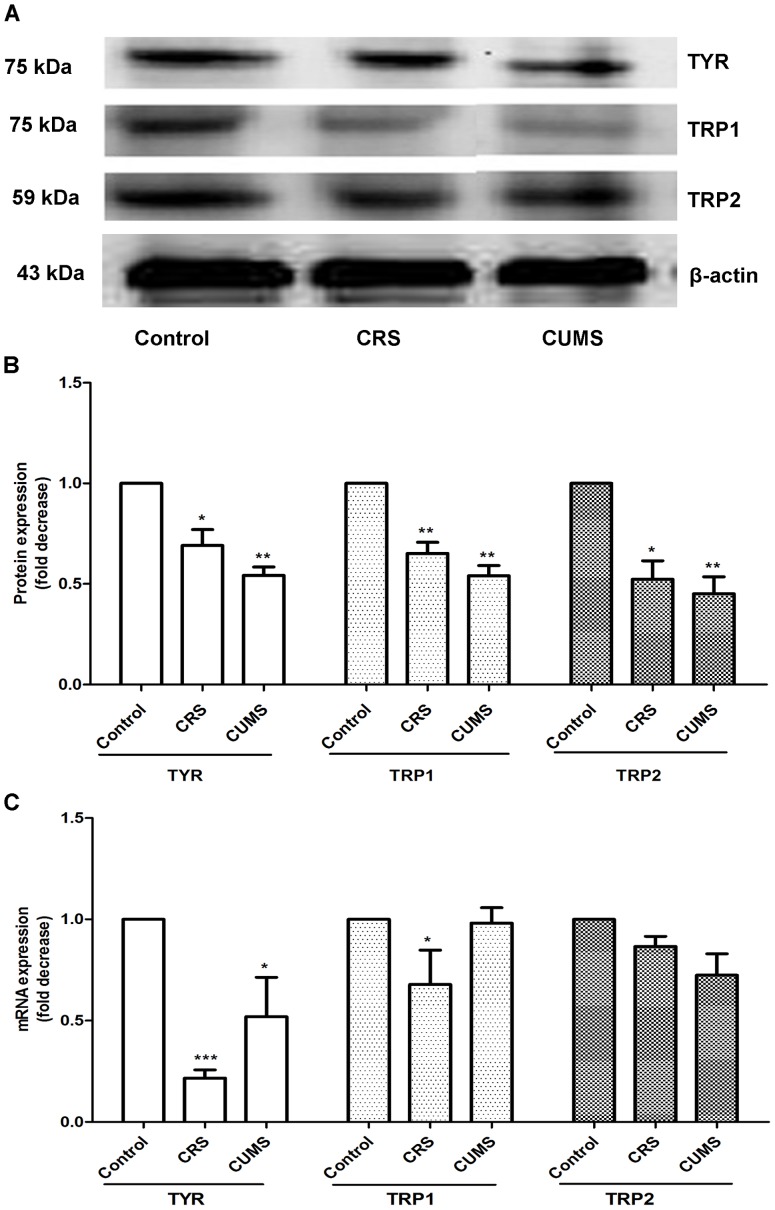
Effect of stress on the expression of TYR, TRP1 and TRP2 in mice dorsal skin. A: Representative Western blot analysis of TYR, TRP1 and TRP2 after stress. B: Quantitation of Western blot analysis for TYR, TRP1 and TRP2. C: The relative expression of TYR, TRP1 and TRP2 was quantified by q-PCR in the dosal skin of nonstressed or stressed animals. Data are expressed as means ± SD (n = 3). *P<0.05, **P<0.01 and ***P<0.001 vs control group.

To further explore molecular mechanisms involved in the whitening of stressed skin, the expression of important regulators of melanogenesis (TYR, TRP1 and TRP2) [28] was compared between nonstressed and stressed mice. By RT-PCR and Western blot analysis, the expression levels of TYR, TRP1 and TRP2 transcript and protein were strongly decreased in stressed skin (Figure 2). At the same time, a significant decrease in the expression of TYR and TRP1 mRNA was seen in the dosal skin of CRS mice compared with CUMS mice, whereas the expression of their proteins was slightly increased (Figure 2). These results indicate that stress impacts on the enzymatic key control of melanogenesis by inhibiting gene transcription and protein expression of TYR, TRP1 and TRP2.

### Alterations of 5-HT Levels in Serum and Skin and *In Vitro* Direct Effects of 5-HT on Pigmentation

**Figure 3:**
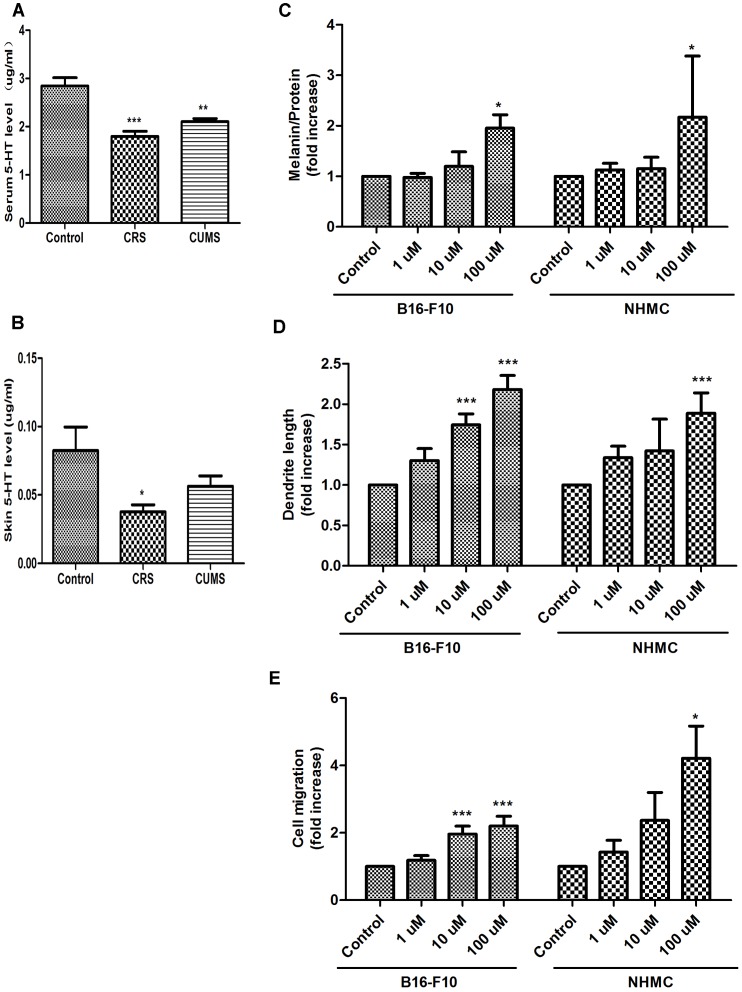
Effect of stress on 5-HT levels and the direct effect of 5-HT on the morphology and behavior of B16F10 cells and NHMCs. Serum (A) and cutaneous (B) 5-HT levels under control, CRS and CUMS conditions. Values represent the mean of 15 animals (± SD). (C, D) B16F10 cells or NHMC cells were cultured in six-well plates without or with various amounts of 5-HT (1, 10 and 100 µmol/L) for 72 h. E: The bottom chamber was filled with MCDB153 and DMEM supplemented with different concentrations of 5-HT. After 24 h, the migrated NHEMs and B16F10 cells were quantified. Results shown are means ± SD (i.e. n = 4). Data are analyzed by one-way analysis of variance (ANOVA) followed by post hoc Turkey test. *P<*0.05*, **P<0.01 and ***P<0.001 vs control group.

To relate 5-HT to stress-induced depigmentary processing, we first analyzed the effect of stress on 5-HT levels in serum and skin. Using LC-MS/MS method, serum 5-HT levels were decreased in both CRS and CUMS mice (Figure 3A). Interestingly, restraint stress alone significantly reduced skin 5-HT levels (Figure 3B). Next, to determine the direct effects of 5-HT on melanogenesis, migration and dendricity, we added 5-HT (1µM ∼100 µM) to the culture medium of B16F10 cells and NHMCs. These cells treated with 5-HT (100 µM) showed a strongly stimulatory effect on melanin synthesis and dendritic network (Figure 3C, 3D). Also, 5-HT could stimulate the migration of B16F10 cells and NHMCs (Figure 3E). These data demonstrated that 5-HT levels were decreased related to stress and could alter morphology and behavior of B16F10 cells and NHMCs, enough to influence the pigmentary processing.

### Effect of Stress on the Expression of Cutaneous 5-HTR System

**Figure 4:**
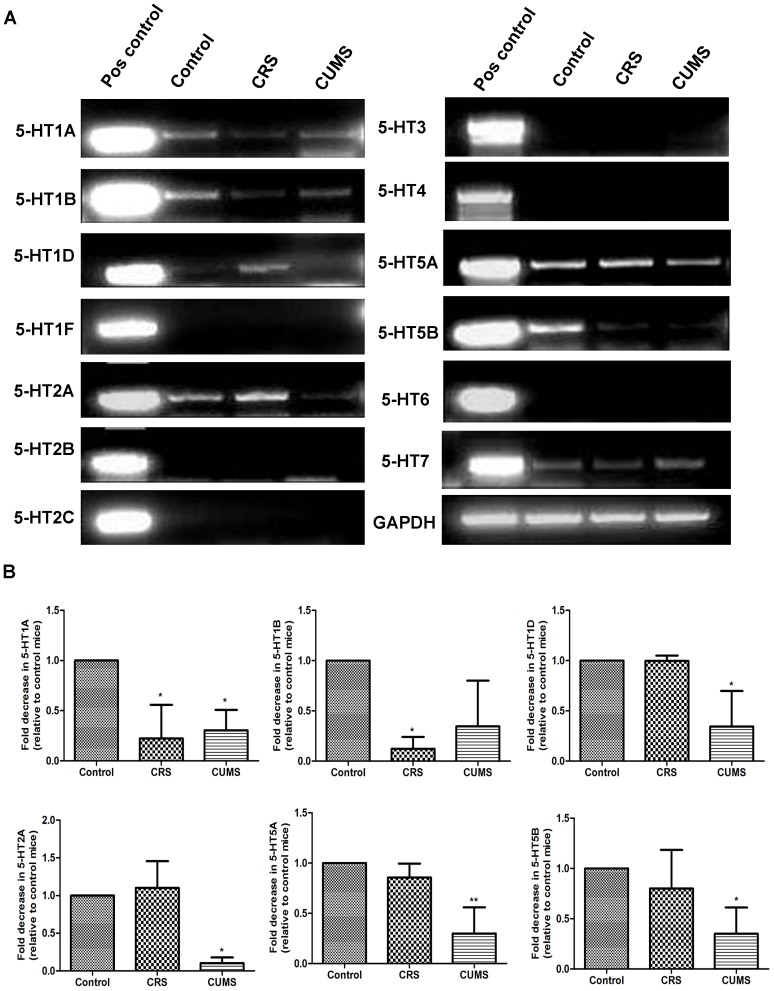
Effect of stress on the expression of skin 5-HTRs. A: Gene expression of the 5-HT1-7 receptor subfamilies. Comparable quantities of cDNA were ensured by amplification of GAPDH. Data are representative of 3 independent experiments. B: The relative expression of 5-HT1A, 1B, 1D, 2A, 5A, 5B and 7 receptors was quantified by q-PCR. Data are means ± SD (n = 2, *P<0.05 and **P<0.01 vs control group).

In order to determine if stress regulates 5-HTR expression, a comprehensive analysis of cutaneous 5-HTR gene expression was performed. RT-PCR and Q-PCR results collectively revealed that the expression of 5-HT1A, 1B, 1D, 2A, 5A and 5B mRNA in stressed skin was down-regulated (Figure 4A). However, 5-HTR7 transcript levels presented no changes between each sample (Figure 4A). Because 5-HTRs are located in all kinds of cells in the mammalian skin [27], 5-HT7 receptor expression in the follicular melanocytes may be changed after CRS or CUMS. Thus, these findings suggest that the observed associations of serotonin with dorsal depigmentation may be mediated by 5-HTR1A, 1B, 1D, 2A, 5A, 5B and 7.

### Effect of 5-HT on Expression of Pigmentation-related Gene and Activation of 5-HTR in Cultured HFs

**Figure 5:**
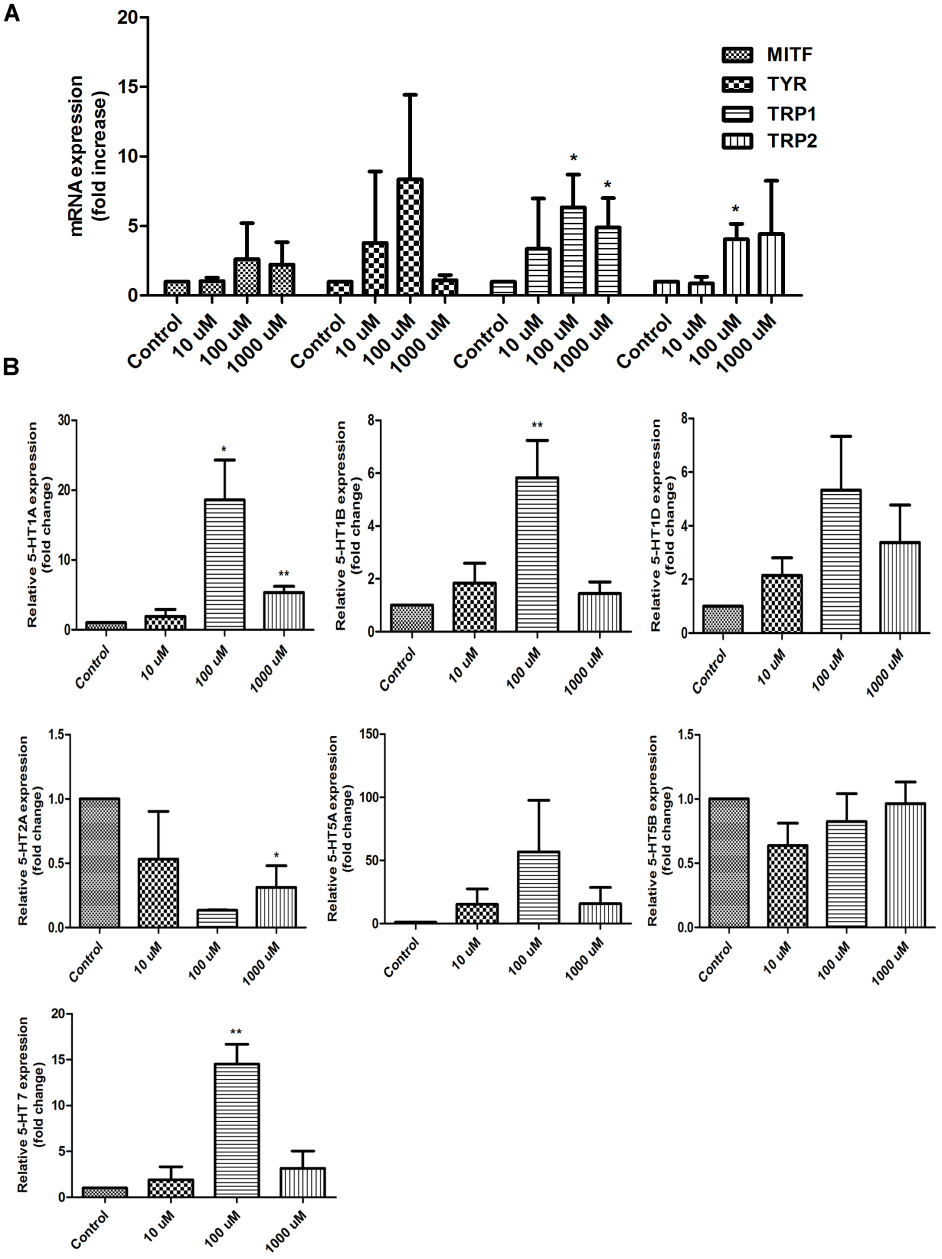
Effect of 5-HT on the expression of MITF, TYR, TRPs and 5-HTRs in cultured HFs. A: mRNA expression analysis of MITF, TYR, TRP1 and TRP2 in 5-HT-treated HFs for 7 days. B: Quantitative results for RT-PCR amplification of 5-HTRs, 1A, 1B 1D, 2A, 5A, 5B and 7 following 5-HT-treated concentration at 10 µM, 100 µM and 1000 µM. Quantities were normalized to endogenous β-actin expression. Data are means ± SD (n = 3, *P<0.05 and **P<0.01 vs control group).

5-HT receptors are also expressed on the HFs [29]. We asked whether 5-HT can improve the pigmentary function of microdissected and organ cultured mice HFs. Quantitative PCR revealed that 5-HT (10–1000 µM) stimulated TRP1 and TRP2 genes transcription compared with vehicle controls (Figure 5A). Microphthalmia transcription factor (MITF) is the most important transcription factor in the regulation of TYR, TRP1 and TRP2 expression [53]. However, the mRNA expression of MITF and TYR was found to remain unchanged (Figure 5A). Moreover, 5-HT could significantly up-regulate the expression of 5-HTR1A, 1B and 7 (Figure 5B). These results imply that 5-HT enhances melanogenesis-related gene transcription, along with the activation of the corresponding receptors (5-HT1A, 1B and 7).

### 5-HT Receptor Involved in Melanogenesis in B16F10 Cells

**Figure 6:**
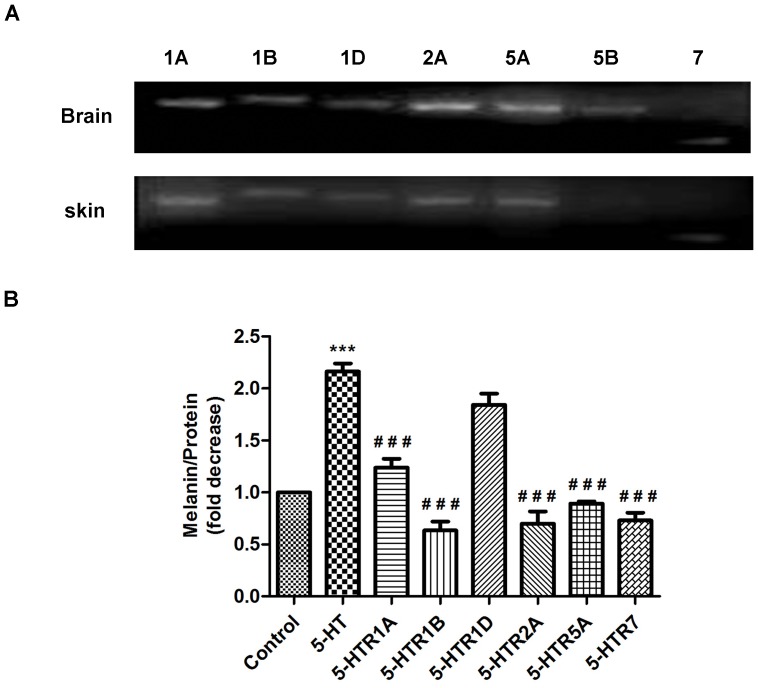
Effect of 5-HTR antagonists (1A, 1B, 1D, 2A, 5A and 7) on 5-HT-induced melanogenesis. A: Representative expression analysis of 5-HTRs transcripts in B16F10 cell and brain (positive control). B: Analysis of 5-HTR1A, 1B, 1D, 2A, 5A and 7 antagonists on 5-HT-mediated melanogenesis. Data are expressed as means ± SD (n = 4). ***p<0.001 vs Control, *###p*<0.001 vs 5-HT (100 µM).

To explore direct pigmentation-modulatory roles of 5-HT *in vitro*, we first tested for the presence of 5-HT receptors. Using RT-PCR, the expression of 5-HT1A, 1B, 1D, 2A, 5A and 7 was detected in B16F10 cells while 5-HT5B was undetectable (Figure 6A). Accordingly, to verify the possible roles of 5-HTR1A, 1B, 1D, 2A, 5A and 7 in melanogenesis, these antagonists were used in competition experiments. As shown in Figure 6B, melanin content in B16F10 cells treated with 5-HT1D antagonist presented no significant changes. However, 5-HT1A, 1B, 2A, 5A and 7 antagonists could partially decrease melanin synthesis (Figure 6B). These data suggest that 5-HT1A, 1B, 2A, 5A and 7 receptors are involved in direct effects on pigmentation.

### *In Vitro* Effect of Serum Obtained from Stressed Mice and 5-HTR1A, 1B and 7 Antagonists on Tyrosinase Activity in Cultured Human Melanocytes

**Figure 7:**
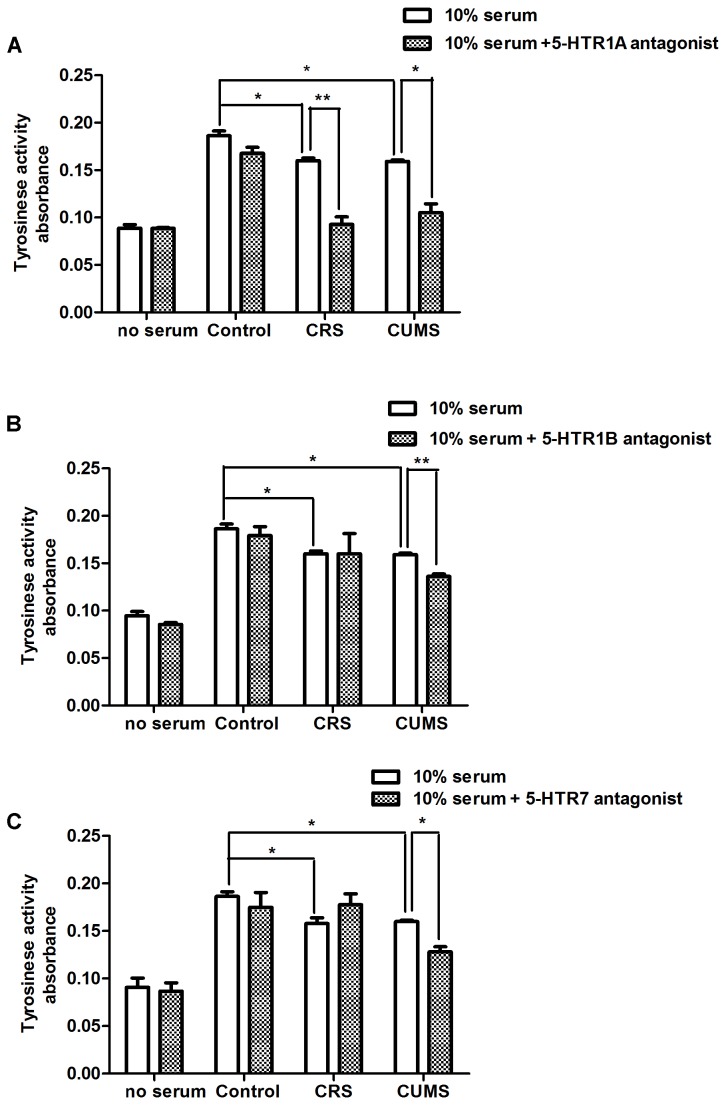
Effect of serum obtained from stressed animals and 5-HTR1A, 1B and 7 antagonists on tyrosinase activity in cultured human melanocytes. A: Effect of serum obtained from stressed animals in the presence or absence of 5-HT1A antagonist (WAY100635) on tyrosinase activity. B: Effects of serum obtained from stressed animals in the presence or absence of 5-HT1B antagonist (SB216641) on tyrosinase activity. C: Effect of serum obtained from stressed animals in the presence or absence of 5-HT7 antagonist (SB269970) on tyrosinase activity. Data are expressed as means ± SD (n = 6). *p<0.05, **p<0.01.

Serum was obtained from animals undergoing CRS and CUMS to examine the direct effects of the serum factor on tyrosinase activity of human melanocytes. Although 5-HT2A and 5-HT5A could be responsible for melanogenesis in B16F10 cells (Figure 6), they were not apparently activated by 5-HT in cultured HFs (Figure 5B). Thus, we added 5-HTR1A, 1B and 7 antagonists to the culture medium of melanocytes at serial dilution. Then, the serum obtained from stressed mice showed significantly decreased tyrosinase activity in NHMCs compared to that from nonstressed mice (Figure 7). It was also found that preincubation with 5-HT1A, 1B and 7 antagonists could further attenuate tyrosinase activity (Figure 7).

### Pigmentary Responses of 5-HTP, FX and 5-HTR1A/1B Agonists to Stressed Mice

**Figure 8:**
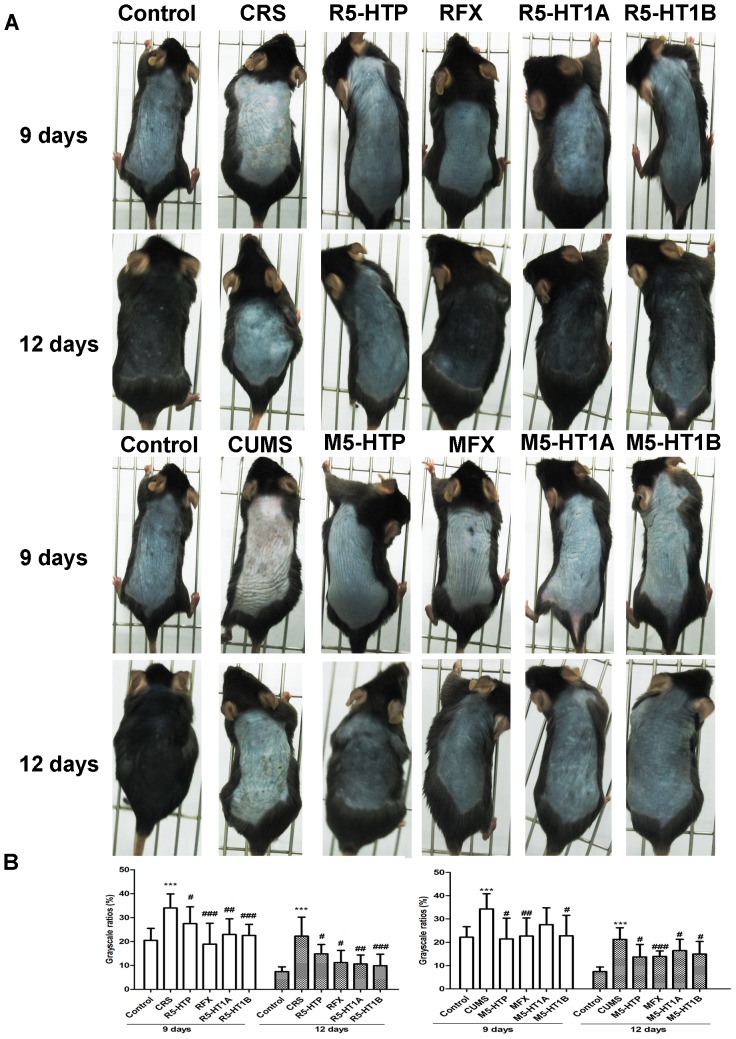
Macroscopic observations of the pigmentary response by administration of 5-HTP, FX and 5-HT1A/1B agonists to stressed mice. A: The significant area of color in the dorsal skin was from neck to tail. B: The corresponding skin color gray-scale ratio on day 9 and day 12 was shown. Data are presented as mean ± SD, n = 10 in each group, ***P<0.001 vs control group, *#p*<0.05 vs CRS or CUMS group, *##p*<0.01 vs CRS or CUMS, *###p*<0.001 vs CRS or CUMS group.

It is possible that 5-HT1A, 5-HT1B and 5-HT7 receptors are directly involved in stress-induced depigmentation. Nevertheless, the expression of 5-HT7 receptor in overall skin showed no change (Figure 4A). Consequently, to better understand the role of 5-HT-led signaling on stress-induced depigmentary damage, 5-HTP, FX and 5-HTR1A/1B agonists were administered into stressed mice. As shown in Figure 8, the treatment with 5-HTP, FX and 5-HTR1A/1B agonists resulted in restoration of the dorsal depigmentation in stressed mice. The skin color with FX base treatment to stressed mice was unchanged compared with the model groups (data not shown). In addition, to verify the role of 5-HT in pigmentation again, depletion of 5-HT in stressed animals was performed by tryptophan-free diets (TFD) for three weeks. A three week tryptophan restriction significantly reduced the concentration of serotonin in brain and blood [54], [55]. The result showed that the depletion of 5-HT obviously aggravated depigmentary damage in stressed skin (Figure S1). These results collectively suggest that the depigmentary damage in stressed mice may be effectively restored by administration of 5-HTP, FX and 5-HT1A/1B receptor agonists.

## Discussion

This study demonstrated that two types of stress caused skin depigmentary response in mice. This phenomenon is concordant with premature graying in humans, who are confronted with increasing pressure in the current world. Increasing, experimental studies also suggest a role for stress-induced signaling in mediating various effects on skin pigmentation function. Psychoemotional stress has an inhibitory effect on the hair growth in mice [12]. However, in HR-1 × HR/De F1 female mice, stress does not affect the development of pigmentation nor change the number of DOPA-positive melanocytes [56]. Our results here showed that stress could attenuate the follicular melanogenesis, thus leading to the dorsal whitening in C57BL/6 mice (Figure 1A). On day 9 after depilation, stresed mice obviously failed to produce fully pigmented hair. This depigmentary response became striking on day 12 after depilation (Figure 1A). Then, we harvested skin samples for HE assay when most of hair follicles from three groups did not entered the catagen phase. Using HE assay, macroscopic observations showed that follicular melanin granules were decreased in stressed skin (Figure 1C), resulting in the dorsal whitening. At this point of time, skin in control mice became fully pigmented. The inconsistent findings to previous studies suggest that the link between stress and pigmentary response may be complex. This discrepancy may be due to the conditions of stress or the species of animal. Next, Western blot and PCR results indicated that stress influenced the melanin production through the down-regulation of TYR, TRP1 and TRP2 transcription and expression, which are master regulators of pigmentation [28]. Also, CRS led to a decrease on TYR and TRP1 mRNA expression, whereas a sustained increase in TYR and TRP1 protein expression compared to CUMS (Figure 2A, 2B). The data implied that TYR and TRP1 expression might be subjected to post-transcriptional regulation.

External psychological or physical danger (stress) stimulates the animal (human) to respond both consciously and unconsciously [1]. Multiple levels of systems are present that preserve homeostasis. Neuropeptides and neurotransmitters produced and released in response to stress, produce profound effects on skin pigmentary function [28]. Similarly, serotonin that has key functions in the central and peripheral nervous systems can exert pigment modulatory effects [28]. Emerging data also demonstrate that serotonin can be transported or released by skin cells [27]. In our previous study, it was found that FX could enhance melanogenesis in B16F10 cells and NHMCs [32]. We have now extended this initial study to examine 5-HT signaling in pigmentary responses related to stress and reveal novel functions. First, we showed that 5-HT levels in serum and skin were decreased following stress (Figure 3A, 3B). Synthesis and secretion of serotonin can be influenced by steroids, neuropeptides and growth factors [27]. Stress activates the HPA axis, which results in elevated levels of circulating glucocorticoids, that stimulate the synthesis and turnover of 5-HT [57], [58]. In skin, 5-HT is synthesized and released by epidermal melanocytes, Merkel cells (cells that receive many axon terminals) and inflammatory cells such as mast cells [27]. Therefore, changes of 5-HT levels appear to be associated with various pathways under stressful condition. Second, alterations in the levels of 5-HT may alter the maturation, metabolism, migration and mitosis of its target cells, including those in both the brain and the skin [27]. Thus, we added 5-HT to the culture medium of skin melanocytes and B16F10 cells, finally exerting positive effects on the morphology and behavior of these cells (Figures 3C, 3D and 3E). This provides direct evidence that 5-HT is implicated in pigmentation *in vitro*, similar to that found in other pigment cells [31], [59]. These findings suggested that 5-HT showed a functional consistency in behavior and morphology of melanocyte. In addition, the content variation of 5-HT in stressed mice implied important functions for serotonin. Abnormalities in 5-HT levels are likely to be linked to stress-induced hypopigmentation.

The mammalian skin cells have the capability to produce and metabolize serotonin. The cutaneous phenotypic effects are mediated by its interactions with 5-HT receptors [19]. As shown in Figure 3A, it was evident that levels of 5-HTR expression were down-regulated in connection with stress. 5-HTR1A levels decreased in both CRS and CUMS mice, a phenomenon that is either because of direct action of cortisol on gene transcription [60] and/or feedback inhibition [61]. Chronic stress may have impacts on the skin barrier, thereby worsening skin diseases [62]. As 5-HTR1A is expressed in the outer part of the epidermis [27], alterations of this receptors in chronic stress may modulate the protective function of this barrier. Moreover, serotonin receptors are also expressed on sensory nerve endings, which transmit to the brain information on changes on skin scratching and dermatitis induced by either intrinsic or environmental factors [8]. Certain evidence indicates that these receptors are also involved in pigmentary functions. For example, cultures of skin and skin cells express receptors for 5-HT [27]. Additional investigations in skin have demonstrated expression of 5-HTR1A by basal epidermal melanocytes and of 5-HTR2A in the epidermis [63]. Recently, the findings have demonstrated that serotonin can affect pigmentation by its interactions with 5-HT receptors in melanomas, NHMCs and frog melanophores [31], [59]. Therefore, the possible role for 5-HTR1A, 1B, 1D, 2A, 5A, 5B and 7 in stress-induced hypopigmention, should be also suggested.

Because the skin truncal melanocytes are confined to the hair follicle and the intrafollicular melanogenesis exclusively reflects the skin color in C57BL/6 mice [36], [37], we wanted to investigate whether 5-HT could induce the expression of the melanogenesis-related genes in cultured hair follicle organ. Through Q-PCR assay, our HF organ culture data had clearly shown that the expression of TRP1 and TRP2 was significantly increased in 5-HT-treated HFs compared with vehicle controls (Figure 5A). Meanwhile, it was accompanied with enhanced expression of 5-HT1A, 5-HT1B and 5-HT7 receptors in the HFs (Figure 5B). Although the mRNA expression of 5-HT7 receptor in stressed skin presented on changes (Figure 4), this receptor expression in the follicular melanocytes may be changed after CRS or CUMS. Thus, our study was also to determine a direct role of 5-HT7 receptor in melanogenesis. Next, to further address a direct role of 5-HTRs in pigmentation, we added 5-HT and its antagonists to the culture medium of B16F10 cells. The result showed 5-HTR1A, 1B and 7 antagonists could partially block 5-HT-induced melanogenesis. This finding was somewhat inconsistent with the previous report that 5-HT1 receptor agonists can fail to enhance melanogenesis [31]. This discrepancy may be attributed to the different biochemical and pharmacological profiles between 5-HT1 receptor antagonist and agonist or the different melanoma cell lines. Combined, 5-HT promoted melanin synthesis probably through the activation of 5-HT1A, 1B and 7.

St John’s Wort and Syrian rue have used alternative plant medicine to improve depigmentation in vitiligo and both of them evoke 5-HT syndromes [64]. Therefore, these plants may be used in vitiligo due to their actions related to 5-HT. Patients with inherited vitiligo have been shown to have significantly lower TPH1 expression, leading to low serotonin levels after Epidermal H_2_O_2_/ONOO(-)-mediated stress [65]. Chronic restraint is reported to induce oxidative stress in many organs [66]. Thus, CRS might induce oxidative stress, leading to declining serum and skin 5-HT levels (Figure 3A, 3B). Low serotonin levels are assumed to be responsible for depigmentation in stressed skin. Though decreased 5-HT levels of serum and skin may not play a direct role in depigmentation, it is possible that 5-HT acts as a factor that attenuates dorsal pigmentary responses in stressed mice. As shown in Figure 7, *in vitro*, the serum obtained from stressed animals significantly decreased tyrosinase activity in human melanocytes compared with nonstressed animals. Then, it was also found that preincubation with 5-HTR1A, 1B and 7 antagonists could further attenuate tyrosinase activity (Figure 7). These data collectively showed that 5-HT was also effective on the pigmentation of melanocytes in culture. This action of 5-HT confirmed that 5-HTR1A, 1B and 7 were expressed in skin melanocytes. This is consistent with detection of 5-HT1A, 5-HT1B and 5-HT7 receptors on mammalian melanocytes [27]. On the other hand, stress is related to elevated corticosterone levels in both plasma and skin as a result of HPA axis activation [67], [68]. Chronic stress as a result of the hypersecretion of corticosterone, initiates a cascade of changes with impact on 5-HT system [15]. There has been a report that pretreatment with corticostatin was effective in inhibiting the pigmentary response in stressed mice [56]. Thus, our further investigation of the 5-HT system, their signal transduction in melanocytes, and the functional consequences of their interaction with HPA axis needs to be improved. However, our data indicate that the decrease in circulating 5-HT levels may be at least partially responsible for the attenuation of pigmentary responses under stressful conditions.

5-HTP can cause dose-related increases in 5-HT [69], [70] and FX increases 5-HT levels in the synapse at the presynaptic neuron. FX can cause effects associated with the increased serotoninergic response [71], [72]. An increased 5-HT concentration leads to increased activation of 5-HT receptors [73]. Meanwhile, 5-HTP and FX have been reported to stimulate 5-HT transmission [74]. To evaluate possible roles of 5-HT *in vivo*, we treated 5-HTP and FX with increasing amounts of 5-HT and 5-HT1A/1B receptor agonists with the activation of 5-HT1A/1B receptors [75]. Our results showed that treatment with 5-HTP, FX and 5-HT1A/1B receptor agonists could prevent dorsal whitening in stressed mice (Figure 8), thus confirming the undoubted role of 5-HT-5-HT1A/1B receptors in stress-induced abnormal pigmentary responses. Our finding also revealed that levels of 5-HT were decreased in skin of stressed mice (Figure 3B). 5-HT was important for the development of morphology and behavior of melanocytes (Figure 3C) [31] and it serves as a regulator in modulating the release of ACTH and α-MSH [16], [17], which fluctuate considerably in adults melanocytes as a function of MC-1R-dependent activation of the cAMP pathway [28]. Previous *in vitro* studies have suggested that 5-HT-induced physiological effects are mediated via distinct classes of receptors, which possibly participate in the modulation of pigmentary responses [31], [59]. Our data demonstrated that this effect presented *in vivo*. The increase of 5-HT levels ameliorated depigmentary damages (Figure 8). This damage could also be partially reversed by 5-HT1A/1B receptor agonists (Figure 8). Conversely, depletion of 5-HT from brain and blood had some serious consequences on pigmentary responses (Figure S1). Obviously, there is growing evidence that 5-HT is playing an important role of pigmentary responses in the setting of stress. Dysfunction of 5-HT-5-HT1A/1B receptors signaling can lead to or/and be a marker of stress-induced hypopigmentation disorders. The consistency of the phenotypes reported here also lend confidence to the validity of serotonin associations and support the theory that serotonin contributes to the development of hair growth and pigmentation via 5-HT1A/1B receptors. Unfortunately, despite an extensive amount of information concerning serotonin and its receptors, no drugs have been developed to date to address skin pigmentary disorders. Further investigation of the functions and dysfunctions of the serotonin system will undoubtedly lead to novel treatments of skin hypopigmentation, especially those worsened with stress.

## Supporting Information

Figure S1 **Macroscopic observations of the pigmentary response after depletion of 5-HT in stressed animals.** RTFD group: application of CRS concomitant with tryptophan-free diets for three weeks; MTFD group: application of CUMS concomitant with tryptophan-free diets for three weeks.
(TIF)
