# Supplementary figures and images for: 5-HT1A/1B Receptors as Targets for Optimizing Pigmentary Responses in C57BL/6 Mouse Skin to Stress
Source: PLoS One. 2014 Feb 21;9(2):e89663. doi: 10.1371/journal.pone.0089663 (PMC3931828; doi:10.1371/journal.pone.0089663)

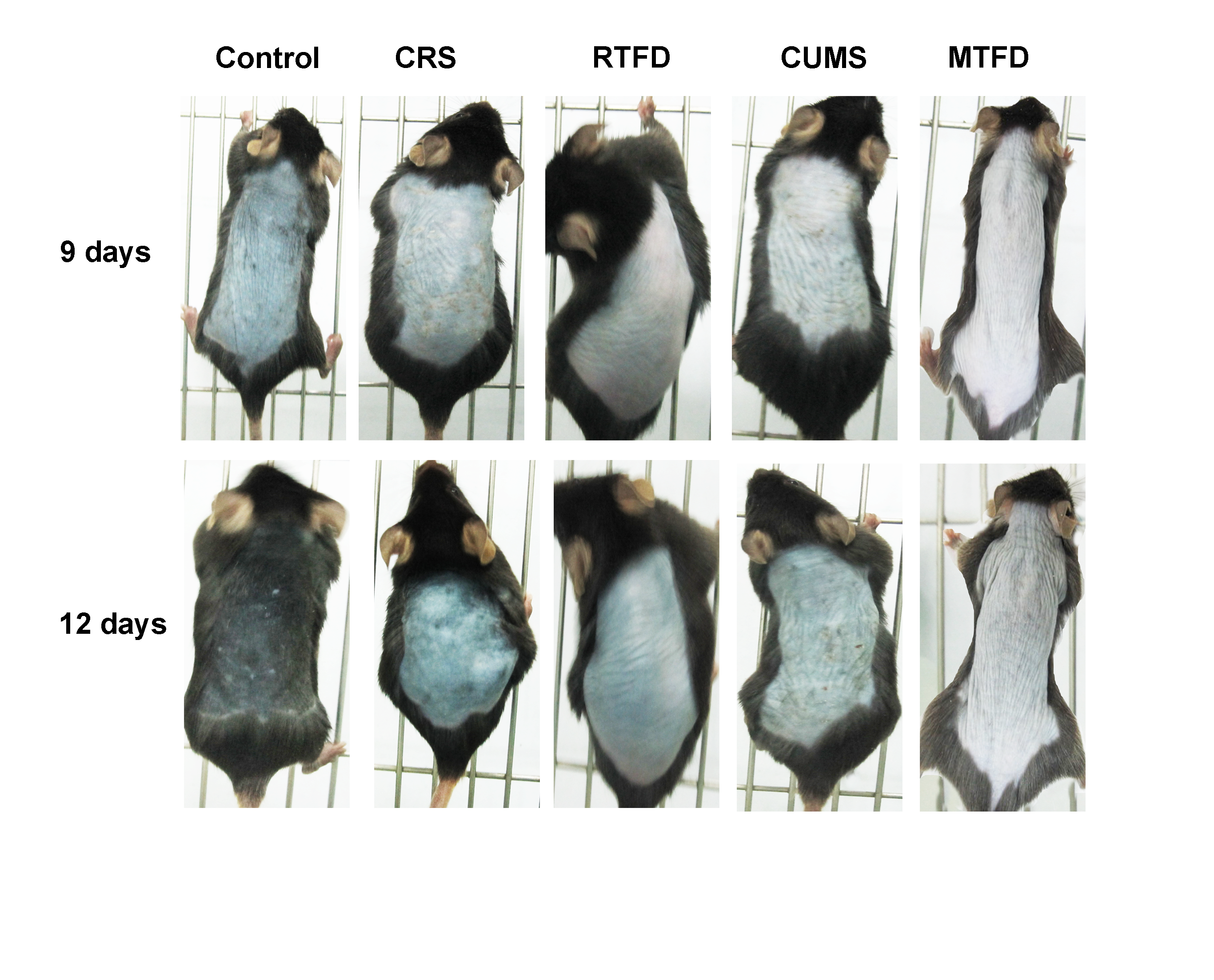

Supplement: Figure S1 — Macroscopic observations of the pigmentary response after depletion of 5-HT in stressed animals. RTFD group: application of CRS concomitant with tryptophan-free diets for three weeks; MTFD group: application of CUMS concomitant with tryptophan-free diets for three weeks. (TIF) [file pone.0089663.s001.tif]
